# Successful Passage per Rectum of a Key

**Published:** 1910-07-09

**Authors:** G. Ainsworth


### The General Practitioner's Column.

**[Contributions to this Column are invited, and, if accepted, will be paid for.]**

### SUCCESSFUL PASSAGE PER RECTUM OF A KEY.

By G. AINSWORTH, M.B., Ch.B.

A short time ago the writer had an interesting case, which from its sequel is worth relating. The patient was a male child of two years of age. I was sent for in great haste one night with the information that the child had swallowed a key of fair dimensions. The fact that the key was slightly rusty and an awkward shape from the point of view of a successful recovery further increased the parents' and my anxiety as to the result. At first sight there was nothing particularly disquieting in the appearance of the boy. He was pale, quieter than usual, and inclined to drowsiness. Temperature was normal, the pulse was rather quicker and not so forcible, the skin was clammy, and pupil dilated. On palpating the abdomen, tenderness in two places seemed evident, otherwise the abdominal wall was flaccid and soft. On percussion slight dulness was noticed in the region of the cæcum. An emetic of ipecacuanha wine and warm water was first tried with no results, strengthening the theory already formed that the key had gone through the stomach into the intestines. A sedative of bromide of potassium was given to rest the child for the night, and in the absence of urgent symptoms I decided to await results. The following morning I ordered a purgative to be given early, and porridge to be the only food for breakfast. The next day I was still agreeably surprised to note the child seemed little, if any, the worse for its experience. The bowel was further cleared by an enema of warm water and soap, but nothing was obtained except slimy motion. The child had had a good night's rest, and seemed free from pain. The porridge diet was continued the next day and the castor oil the following morning, followed by another warm injection. This process was continued until the fourth day; the patient meanwhile was not apparently suffering, and no undue nausea or distension occurred. The same evening I received a visit from the father who was in good humour, and to my astonishment informed me the key had been passed in the last motion, and that the child was doing well. He brought the key with him for inspection, and I was surprised at its shape and size. It was approximately 1½ inch in length and ¾-inch in width at its widest part. Its passage was accompanied with very little pain by all accounts, and the youngster was in better spirits. He had a good night's rest, and I found him next morning quite normal and taking food with avidity. Convalescence was uninterrupted.

The remarkable points of interest in this case are the absence of pain or distension, the freedom from acute symptoms of bowel obstruction, such as vomiting, peritonitis, and subnormal temperature, and the good recovery without recourse to operation.

In cases such as this there may be constipation (temporary and complete) or watery diarrhœa, pain, colic, vomiting, and distension. Sometimes one can feel the fæces or tumour, and dulness is noticed on percussion. Should chronic obstruction supervene a common sign is peristalsis—movements of the distended coils of intestine.

